# A Rare Pediatric Intraductal Papillary Mucinous Neoplasm Presenting as a Pancreatic Pseudocyst With Splenic Vein Thrombosis and Gastric Variceal Hemorrhage

**DOI:** 10.7759/cureus.111084

**Published:** 2026-06-18

**Authors:** Razan A Hassan, Mafaz Ombada, Salik Taufiq

**Affiliations:** 1 Pediatrics, University of Florida College of Medicine – Jacksonville, Jacksonville, USA; 2 Pediatric Gastroenterology, Nemours Children's Health System, Jacksonville, USA

**Keywords:** branch-duct ipmn, gastric varices, intraductal papillary mucinous neoplasm, ipmn, pancreatic cyst, pancreatic neoplasm, pancreatic pseudocyst, pediatric pancreas, sinistral portal hypertension, splenic vein thrombosis

## Abstract

Intraductal papillary mucinous neoplasm (IPMN) is a rare pancreatic cystic neoplasm in children. Pediatric pancreatic cystic lesions are more often inflammatory, traumatic, or congenital; therefore, IPMN may be mistaken for pseudocysts or pancreatitis-related changes.

We report the case of a 15-year-old girl who initially presented with intermittent abdominal pain, mild lipase elevation, and a pancreatic tail cyst that was managed as a presumed pseudocyst after interval regression on surveillance imaging. Despite initial improvement, she later developed upper gastrointestinal bleeding. Subsequent evaluation demonstrated progressive cyst enlargement, splenic vein thrombosis, splenomegaly, collateral vessel formation, and isolated gastric varices consistent with sinistral portal hypertension.

Ultrasound and cross-sectional imaging confirmed progression of the pancreatic lesion, and endoscopic ultrasound with biopsy established the diagnosis of branch-duct IPMN. Following immunization against encapsulated organisms, the patient underwent robotic-assisted distal pancreatectomy with splenectomy. Surgical pathology confirmed a large branch-duct IPMN without invasive carcinoma. Recovery was uncomplicated, and follow-up showed no evidence of recurrence.

This case highlights the diagnostic challenges of pediatric IPMN, particularly when it mimics a pancreatic pseudocyst and demonstrates apparent interval regression. It also illustrates the potential for significant vascular complications, including splenic vein thrombosis, sinistral portal hypertension, and gastric variceal hemorrhage. Persistent or evolving pancreatic cystic lesions in children warrant ongoing reassessment, especially when accompanied by vascular abnormalities. Early recognition and definitive management may prevent substantial morbidity and facilitate timely treatment of this rare pediatric pancreatic neoplasm.

## Introduction

Intraductal papillary mucinous neoplasms (IPMNs) are mucin-producing epithelial neoplasms arising from the pancreatic ductal system and are recognized precursor lesions to pancreatic adenocarcinoma. IPMNs are classified as main-duct, branch-duct, or mixed-type lesions and are well described in adults, where management strategies are guided by the risk of malignant transformation and progression to invasive carcinoma [1].

In contrast, IPMN is exceedingly rare in children, and the natural history, diagnostic approach, and optimal management of pediatric IPMN remain poorly defined. Published pediatric experience with IPMN is limited to a small number of case reports and case series. Reported cases include a neonate with IPMN and high-grade dysplasia associated with congenital hyperinsulinism and a de novo germline *SKIL* mutation, a 14-year-old boy with main-duct IPMN presenting with recurrent abdominal pain and recurrent pancreatitis, and a 12-year-old boy with branch-duct IPMN identified after recurrent pancreatitis and concerning endoscopic ultrasound findings [2-4]. In addition, a recent 26-year single-center review of pediatric pancreatic disease identified only one case of IPMN among all pediatric pancreatic disorders evaluated [5]. These reports suggest that pediatric IPMN may present across a broad age spectrum and with diverse clinical manifestations, most commonly abdominal pain, pancreatitis, or pancreatic cystic lesions.

We report a 15-year-old girl with branch-duct IPMN initially managed as a presumed pancreatic pseudocyst. Her disease course was characterized by apparent interval regression followed by progressive cyst enlargement, splenic vein thrombosis, sinistral portal hypertension, and gastric variceal hemorrhage. Compared with previously reported pediatric cases, the combination of pseudocyst mimicry, evolving vascular complications, and left-sided portal hypertension appears particularly unusual. This case highlights the diagnostic challenges, radiologic evolution, vascular complications, and surgical management of pediatric IPMN.

## Case presentation

**Figure 1:**
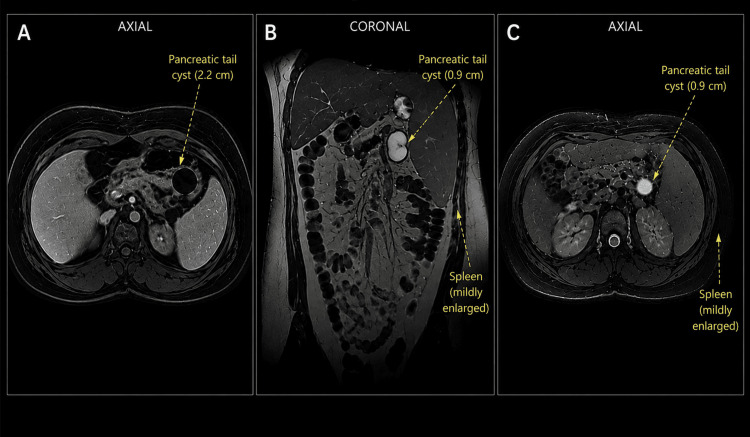
MRI demonstrating the initial pancreatic tail cyst and subsequent interval regression (A) Axial MRI demonstrating a well-circumscribed 2.2-cm cystic lesion in the pancreatic tail without mural nodule, solid component, or restricted diffusion. (B) Coronal follow-up MRI demonstrating interval decrease in cyst size to 9 × 8 mm with mild splenomegaly. (C) Axial follow-up MRI confirming interval regression of the pancreatic tail cyst and persistent mild splenomegaly. No abnormal enhancement, solid components, or restricted diffusion were identified on follow-up imaging. MRI: magnetic resonance imaging

A 15-year-old girl initially presented in August 2023 with intermittent abdominal pain and a mildly elevated serum lipase of 107 units per liter (U/L) (reference range: 0-70 U/L). Magnetic resonance imaging/magnetic resonance cholangiopancreatography (MRI/MRCP) obtained on August 29, 2023, demonstrated a 2.2-cm well-circumscribed cystic lesion in the pancreatic tail without a mural nodule, solid component, pancreatic ductal dilatation, or restricted diffusion (Figure 1). In the setting of intermittent abdominal pain, mildly elevated serum lipase, and the absence of high-risk radiographic features, the lesion was interpreted as a pancreatic pseudocyst versus a benign epithelial cyst. Given the reassuring imaging characteristics, surveillance imaging was recommended.

Follow-up MRI performed on February 29, 2024, demonstrated interval regression of the lesion to 9 × 8 mm without abnormal enhancement, solid components, or restricted diffusion (Figure 1). Mild splenomegaly measuring up to 14 cm was also noted. Given the apparent decrease in size and otherwise reassuring imaging characteristics, conservative observation was continued.

**Figure 2:**
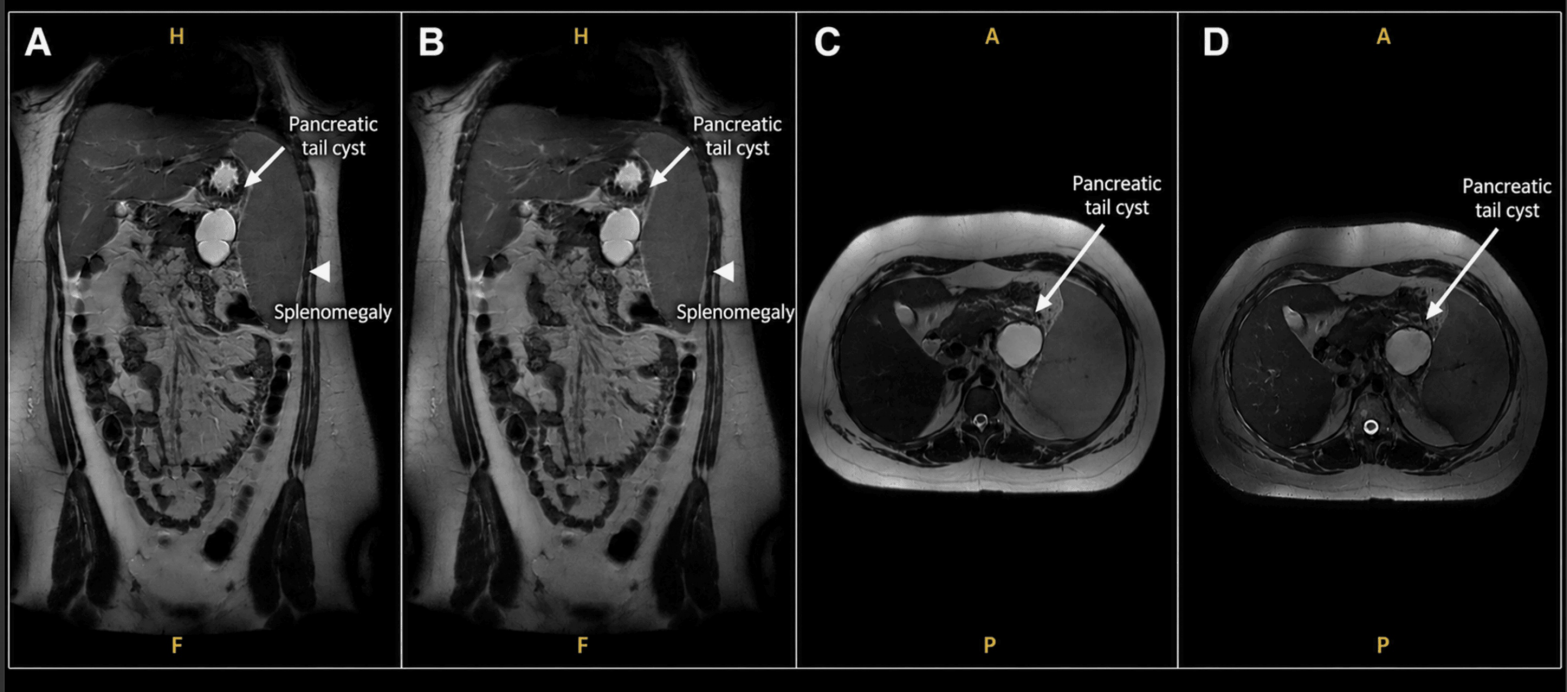
MRI abdomen demonstrating interval enlargement of the pancreatic tail cyst with associated splenomegaly. (A-B) Coronal T2-weighted images demonstrating a lobulated cystic lesion arising from the pancreatic tail (arrow) measuring approximately 5.6 × 4.6 × 4.9 cm. (C-D) Axial T2-weighted images confirming the pancreatic tail cyst (arrow). Associated splenomegaly is visible on both coronal and axial views. MRI: magnetic resonance imaging

In August 2025, the patient developed upper gastrointestinal bleeding. Initial esophagogastroduodenoscopy demonstrated a bleeding gastric ulcer without evidence of gastric varices. MRI of the abdomen and pelvis obtained on August 9, 2025, demonstrated interval enlargement of the pancreatic tail cyst to 5.6 × 4.6 × 4.9 cm with mild internal debris and surrounding peripancreatic inflammatory change (Figure 2). Marked splenomegaly measuring approximately 18 cm and prominent collateral vessels along the splenic hilum and proximal gastric wall were also identified, raising concern for evolving sinistral portal hypertension. Given the patient's history of pancreatitis and associated inflammatory changes, the lesion continued to be favored radiographically as a pancreatic pseudocyst.

**Figure 3:**
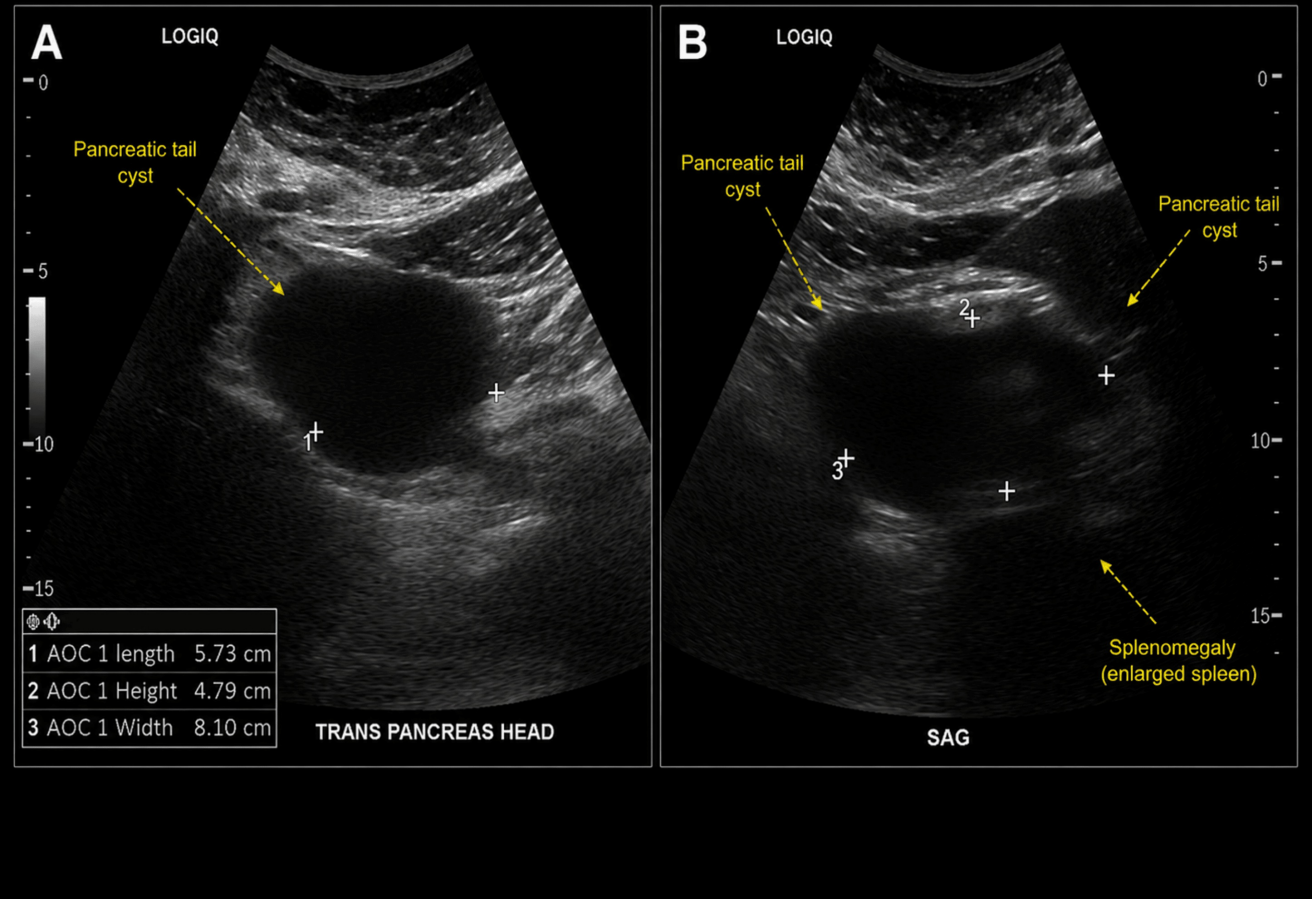
Abdominal ultrasound demonstrating interval enlargement of the pancreatic tail cyst. (A) Transverse ultrasound view demonstrating a large cystic lesion arising from the pancreatic tail with measured dimensions of 8.10 cm × 5.73 cm × 4.79 cm. (B) Sagittal ultrasound view confirming the cystic pancreatic tail lesion.

Doppler ultrasound performed on September 16, 2025, demonstrated further enlargement of the pancreatic tail cyst to approximately 8.0 × 5.7 × 4.8 cm and massive splenomegaly measuring 20.2 cm (Figure 3). The main portal vein remained patent with normal hepatopetal flow, supporting left-sided rather than generalized portal hypertension. Repeat esophagogastroduodenoscopy performed shortly thereafter revealed newly developed isolated gastric varices. Subsequent evaluation confirmed splenic vein thrombosis, consistent with evolving sinistral portal hypertension secondary to pancreatic disease.

Given the progressive enlargement of the lesion and associated vascular complications, additional diagnostic evaluation was pursued. A hereditary pancreatitis panel was negative for pathogenic variants in *PRSS1, CPA1, SPINK1, CTRC, CASR,* and *CFTR*. Endoscopic ultrasound-guided evaluation performed on October 30, 2025, demonstrated cyst fluid carcinoembryonic antigen (CEA) of 723 nanograms per milliliter (ng/mL) and amylase of 25,580 units per liter (U/L). Cytologic evaluation was negative for malignancy but was interpreted as suggestive of IPMN. Collectively, the cyst fluid profile, cytologic findings, and imaging characteristics were consistent with branch-duct IPMN and prompted definitive surgical management.

Following immunization against encapsulated organisms, the patient underwent robotic-assisted distal pancreatectomy with splenectomy on January 2, 2026, approximately two months after endoscopic ultrasound-guided evaluation. Gross examination demonstrated a large cystic pancreatic tail lesion. Histopathologic evaluation confirmed branch-duct IPMN without evidence of invasive carcinoma. The postoperative course was uncomplicated.

At approximately three months postoperatively, an abdominal ultrasound performed on April 1, 2026, demonstrated no recurrent pancreatic lesion, fluid collection, or imaging evidence of acute pancreatitis. The remaining pancreatic parenchyma demonstrated mild echogenic and lobular postoperative changes, and the portal vein remained patent with normal flow. The patient remained clinically well without recurrent pancreatitis or gastrointestinal bleeding during the available follow-up period. A summary of the patient's clinical course is provided in Table 1.

**Table 1:** Clinical timeline of disease evolution and management Chronologic summary of the patient's clinical course, illustrating the progression from an initially presumed pancreatic pseudocyst with interval regression to subsequent cyst enlargement, splenic vein thrombosis, sinistral portal hypertension, gastric variceal hemorrhage, confirmation of branch-duct intraductal papillary mucinous neoplasm (IPMN), and definitive surgical management with distal pancreatectomy and splenectomy. MRI/MRCP: magnetic resonance imaging/magnetic resonance cholangiopancreatography

Event	Date/timeframe	Key findings or intervention
Initial presentation	August 2023	Intermittent abdominal pain and mild lipase elevation prompted evaluation of a pancreatic tail cyst
Initial MRI/MRCP	August 29, 2023	2.2-cm pancreatic tail cyst without solid component, mural nodule, or restricted diffusion; interpreted as pseudocyst versus benign epithelial cyst.
Follow-up MRI	February 29, 2024	Cyst regressed to approximately 9 × 8 mm; mild splenomegaly noted; conservative surveillance continued
First upper gastrointestinal bleed	August 2025	Endoscopy demonstrated a bleeding gastric ulcer without gastric varices
MRI abdomen/pelvis	August 9, 2025	Interval enlargement of the pancreatic tail cyst to approximately 5.6 × 4.6 × 4.9 cm with marked splenomegaly and collateral vessels along the splenic hilum and proximal gastric wall
Repeat endoscopy	August-September, 2025	Newly identified isolated gastric varices consistent with evolving sinistral portal hypertension
Doppler ultrasound	September 16, 2025	Pancreatic tail cyst enlarged to approximately 8.0 × 5.7 × 4.8 cm, massive splenomegaly (20.2 cm), preserved portal venous flow
Splenic vein thrombosis confirmed	September 2025	Findings consistent with evolving sinistral portal hypertension secondary to pancreatic disease
Pancreatitis gene panel	During diagnostic reassessment	No pathogenic variants identified in PRSS1, CPA1, SPINK1, CTRC, CASR, or CFTR
Endoscopic ultrasound with biopsy	Approximately six weeks after varices identified	Branch-duct intraductal papillary mucinous neoplasm confirmed
Surgery	January 2, 2026	Robotic-assisted distal pancreatectomy with splenectomy following preoperative immunization against encapsulated organisms
Final pathology	January 2026	Large branch-duct intraductal papillary mucinous neoplasm without invasive carcinoma
Follow-up	Postoperative interval	No recurrence on imaging; clinically well without recurrent pancreatitis or gastrointestinal bleeding

## Discussion

IPMN is a mucin-producing epithelial neoplasm of the pancreatic ductal system with a biologic spectrum ranging from low-grade dysplasia to invasive carcinoma [1]. Although well recognized in adults, IPMN remains exceedingly uncommon in children and adolescents, with only isolated pediatric cases reported in the literature [2-5]. Published pediatric experience includes neonatal IPMN associated with congenital hyperinsulinism and high-grade dysplasia, branch-duct IPMN with worrisome endoscopic ultrasound features in a 12-year-old child, and main-duct IPMN in a 14-year-old adolescent presenting with recurrent abdominal pain and pancreatitis [2-4]. A recent 26-year single-center review of pediatric pancreatic disease identified only one case of IPMN among all pediatric pancreatic disorders evaluated [5]. Key clinical features of previously published pediatric IPMN cases and comparison with the present case are summarized in Table 2. Consequently, the natural history, optimal surveillance strategies, and management of pediatric IPMN remain poorly defined.

**Table 2:** Published pediatric intraductal papillary mucinous neoplasm cases and comparison with the present case. Summary of published pediatric intraductal papillary mucinous neoplasm (IPMN) cases [2-4] and comparison with the present case. The present case is notable for initial pseudocyst mimicry with interval regression followed by progressive cyst enlargement, splenic vein thrombosis, sinistral portal hypertension, and isolated gastric variceal hemorrhage. ERCP: endoscopic retrograde cholangiopancreatography

Author (year)	Age	Sex	Subtype	Key clinical features	Management/outcome
Jiao et al. (2015) [2]	Neonate	Not reported	IPMN with high-grade dysplasia	Congenital hyperinsulinism; de novo germline *SKIL* mutation; *KRAS* somatic mutation identified in the tumor	Surgical resection; normoglycemia achieved after resection; clinically well at 18-month follow-up
Kang et al. (2020) [3]	12 years	Male	Branch-duct IPMN	Recurrent pancreatitis; pancreatic cystic lesions with worrisome features identified on endoscopic ultrasound	Surgical resection; pathology confirmed branch-duct IPMN
Fanjiang et al. (2007) [4]	14 years	Male	Main-duct IPMN (borderline malignancy)	Recurrent abdominal pain and recurrent pancreatitis; elevated pancreatic enzymes; dilated pancreatic duct and characteristic “fisheye” papilla on ERCP	Pylorus-preserving pancreaticoduodenectomy; pathology confirmed main-duct IPMN without stromal invasion; clinically well without recurrent pancreatitis at >three-year follow-up
Present case	15 years	Female	Branch-duct IPMN	Initially presumed pancreatic pseudocyst with interval regression followed by progressive enlargement; splenic vein thrombosis; sinistral portal hypertension; isolated gastric variceal hemorrhage	Robotic-assisted distal pancreatectomy with splenectomy; pathology confirmed branch-duct IPMN without invasive carcinoma; clinically well without recurrence at follow-up

This case highlights several important diagnostic challenges. The lesion was initially interpreted as a pancreatic pseudocyst because the patient presented with abdominal pain, mild lipase elevation, and imaging findings lacking high-risk features typically associated with neoplasia. Furthermore, interval regression from 2.2 cm to 9 × 8 mm appeared to support a benign inflammatory process. However, subsequent progression demonstrated that apparent regression did not represent disease resolution. In retrospect, mild splenomegaly during the period of apparent cyst regression may have represented an early clue to evolving splenic venous compromise. The development of splenomegaly during surveillance, followed by cyst enlargement and vascular complications, emphasizes that persistent pancreatic cystic lesions in children warrant continued reassessment even when interval imaging appears reassuring. Pediatric pancreatic cystic lesions are more commonly inflammatory, traumatic, or congenital in origin, which may contribute to delayed consideration of neoplastic etiologies such as IPMN [5].

A particularly notable feature of this case was the evolution of splenic vein thrombosis, sinistral portal hypertension, and gastric variceal hemorrhage. Sinistral portal hypertension results from obstruction of splenic venous outflow, leading to increased pressure within the short gastric and gastroepiploic venous systems and subsequent formation of isolated gastric varices. Unlike generalized portal hypertension, portal venous flow is typically preserved, and liver function remains normal. In our patient, progressive splenomegaly, collateral vessel formation, preserved hepatopetal portal venous flow, and the transition from a bleeding gastric ulcer without varices to newly developed isolated gastric varices strongly supported evolving left-sided portal hypertension. The first endoscopy demonstrated a bleeding gastric ulcer without varices. Several weeks later, imaging demonstrated splenic vein thrombosis, splenomegaly, and collateral vessel formation, and repeat endoscopy revealed isolated gastric varices. This stepwise progression strongly supports splenic vein obstruction from adjacent pancreatic disease, leading to sinistral portal hypertension and gastric variceal hemorrhage.

Compared with previously reported pediatric IPMN cases summarized in Table 2, the present case is distinguished by its vascular evolution. Previous pediatric cases primarily presented with congenital hyperinsulinism, recurrent abdominal pain, pancreatitis, or pancreatic cystic lesions without reported splenic vein thrombosis, sinistral portal hypertension, or gastric variceal hemorrhage [2-4]. In contrast, our patient demonstrated progressive splenic vein thrombosis, collateral vessel formation, and isolated gastric varices, which became the major clues prompting reconsideration of the initial diagnosis. To our knowledge, this pattern of stepwise vascular progression has not been emphasized in previous pediatric IPMN reports.

Current management recommendations for IPMN are derived primarily from adult populations. The International evidence-based Kyoto Guidelines for the management of IPMNs of the pancreas provide recommendations for risk stratification and management of pancreatic cystic lesions [6]. Similarly, the European evidence-based guidelines on pancreatic cystic neoplasms recommend consideration of symptoms, cyst growth, mural nodules, and main pancreatic duct involvement when determining management strategies [7]. Although these recommendations cannot be directly extrapolated to children, they provide a useful framework for clinical decision-making. In the present case, progressive cyst enlargement, symptoms, splenic vein thrombosis, marked splenomegaly, collateral vessel formation, gastric variceal hemorrhage, and diagnostic uncertainty justified definitive surgical management. The diagnostic pathway was further supported by endoscopic ultrasound-guided evaluation demonstrating markedly elevated cyst fluid CEA and amylase levels with cytology suggestive of IPMN.

The vascular complications observed in this case are consistent with previously described features of left-sided portal hypertension secondary to splenic vein obstruction [8].

Distal pancreatectomy with splenectomy addressed both the pancreatic neoplasm and the underlying sinistral portal hypertensive circuit responsible for the patient's vascular complications. Histopathology confirmed branch-duct IPMN without invasive carcinoma, demonstrating that substantial local and vascular morbidity may occur even in the absence of malignant transformation.

This case expands the limited literature describing pediatric IPMN and highlights several important clinical lessons. First, IPMN should remain within the differential diagnosis of persistent pancreatic cystic lesions in children and adolescents, even when imaging initially suggests a pseudocyst. Second, apparent interval regression does not necessarily indicate benign behavior and should not preclude continued surveillance when diagnostic uncertainty persists. Finally, a pancreatic cyst that persists or evolves after apparent regression warrants multidisciplinary reassessment, particularly when splenomegaly, splenic vein thrombosis, collateral vessels, or gastric varices develop. Early reconsideration of the diagnosis may prevent delayed recognition and morbidity associated with sinistral portal hypertension.

## Conclusions

IPMN is rare in children and adolescents, but age alone should not exclude the diagnosis. This case demonstrates that pediatric IPMN may initially mimic a pancreatic pseudocyst and may even exhibit apparent interval regression before subsequent progression. Clinicians should reconsider the diagnosis of a presumed pancreatic pseudocyst when a lesion persists, recurs, enlarges, or develops atypical features such as splenomegaly, splenic vein thrombosis, collateral vessel formation, gastric varices, or other unexplained vascular complications. A pancreatic cyst that persists or evolves after apparent regression warrants multidisciplinary reassessment and consideration of advanced diagnostic evaluation. Early recognition of atypical features may facilitate timely diagnosis and intervention, potentially preventing significant morbidity from gastric variceal hemorrhage, sinistral portal hypertension, and other vascular complications.
